# *In vitro* and *in vivo* Study on an Injectable Glycol Chitosan/Dibenzaldehyde-Terminated Polyethylene Glycol Hydrogel in Repairing Articular Cartilage Defects

**DOI:** 10.3389/fbioe.2021.607709

**Published:** 2021-02-16

**Authors:** Jianhua Yang, Xiaoguang Jing, Zimin Wang, Xuejian Liu, Xiaofeng Zhu, Tao Lei, Xu Li, Weimin Guo, Haijun Rao, Mingxue Chen, Kai Luan, Xiang Sui, Yen Wei, Shuyun Liu, Quanyi Guo

**Affiliations:** ^1^Orthopedics Department, Longgang District People’s Hospital of Shenzhen & The Third Affiliated Hospital (Provisional) of The Chinese University of Hong Kong, Shenzhen, China; ^2^The Second Affiliated Hospital of Luohe Medical College, Luohe, China; ^3^Chinese PLA General Hospital, Institute of Orthopedics, Beijing, China; ^4^Department of Orthopedics, Zhengzhou Seventh People’s Hospital, Zhengzhou, China; ^5^School of Medicine, Jiamusi University, Jiamusi, China; ^6^Medical Research Center of Mudanjiang Medical School, Mudanjiang, China; ^7^The Key Laboratory of Bioorganic Phosphorus Chemistry and Chemical Biology (Ministry of Education) Department of Chemistry, Tsinghua University, Beijing, China

**Keywords:** cartilage defect, glycol chitosan hydrogel, polyethylene glycol, repair, tissue engineering

## Abstract

The normal anatomical structure of articular cartilage determines its limited ability to regenerate and repair. Once damaged, it is difficult to repair it by itself. How to realize the regeneration and repair of articular cartilage has always been a big problem for clinicians and researchers. Here, we conducted a comprehensive analysis of the physical properties and cytocompatibility of hydrogels, and evaluated their feasibility as cell carriers for Adipose-derived mesenchymal stem cell (ADSC) transplantation. Concentration-matched hydrogels were co-cultured with ADSCs to confirm ADSC growth in the hydrogel and provide data supporting *in vivo* experiments, which comprised the hydrogel/ADSCs, pure-hydrogel, defect-placement, and positive-control groups. Rat models of articular cartilage defect in the knee joint region was generated, and each treatment was administered on the knee joint cartilage area for each group; in the positive-control group, the joint cavity was surgically opened, without inducing a cartilage defect. The reparative effect of injectable glycol chitosan/dibenzaldehyde-terminated polyethylene glycol (GCS/DF-PEG) hydrogel on injured articular cartilage was evaluated by measuring gross scores and histological score of knee joint articular-cartilage injury in rats after 8 weeks. The 1.5% GCS/2% DF-PEG hydrogels degraded quickly *in vitro*. Then, We perform *in vivo* and *in vitro* experiments to evaluate the feasibility of this material for cartilage repair *in vivo* and *in vitro*.

## Introduction

The normal anatomical structure of articular cartilage determines its abilities for regeneration, although repair is very limited once damage occurs (Rai et al., 2017). Achieving regeneration and repair of articular cartilage has been a major problem for clinicians and researchers. Current clinical treatments for cartilage damage include non-steroidal anti-inflammatory drugs, platelet-rich plasma (Laudy et al., 2015), microfractures, autologous chondrocyte implantation, and osteochondral transplantation techniques (Lynch et al., 2015; Oussedik et al., 2015), but in most cases, the results are unsatisfactory. Curative effects occur when the tissue formed by repair is mostly fibrocartilage, rather than hyaline cartilage.

Cartilage tissue engineering provides a new approach for regenerating and repairing articular cartilage defects (Johnstone et al., 2013). Seed cells, scaffold materials, and bioactive factors are three key elements of cartilage tissue engineering. Adipose-derived mesenchymal stem cells (ADSCs) can differentiate into a wide range of tissues and are easy to culture *in vitro*. Numerous studies have confirmed that ADSCs show strong proliferative capacity, strong regulation of immune function, and multi-directional differentiation potential, making them ideal seed cells for cartilage tissue engineering. Recently, the use of ADSCs for cartilage regeneration and repair has gradually increased, and satisfactory results have been achieved (Kasir et al., 2015; Zhang et al., 2017; Pleumeekers et al., 2018).

Hydrogels are three-dimensional grid scaffolds composed of hydrophilic polymers. They have a high water content and similar properties to the natural cartilage extracellular matrix. Hydrogels are considered very suitable for artificial extracellular matrixes in tissue engineering (Yang et al., 2017). Injectable hydrogels can be gelled *in situ* in the defect site after injection (Liu et al., 2017), which offers the advantage that a simple, minimally invasive injection method can be used to simplify complex implant procedures. Research groups have independently developed and prepared injectable ethylene glycol chitosan/dibenzaldehyde-functionalized (GCS/DF)-polyethylene glycol (PEG) hydrogels using chitosan and PEG as raw materials. A previous study showed that PEG-based hydrogels crosslinked by Schiff base has good injectability and promotes self-healing (Zhang et al., 2011). The team designed injectable GCS/DF-PEG-loaded ADSCs to repair articular cartilage defects and simplify cartilage-repair operations, representing an important technological advance. In this study, we conducted a comprehensive analysis of the physical properties and cytocompatibility of hydrogel materials, and their feasibility for tissue engineering cell carriers was evaluated. The *in vivo* findings of our study of repair should serve as a valuable reference in the development of tissue engineering for the treatment of articular cartilage defect. Mechanical testing showed that the hydrogel elastic modulus increased proportionally with DF-PEG concentration. Proliferation experiments with mesenchymal stem cells in hydrogels showed that higher DF-PEG concentrations correlated with slower cell proliferation. Co-culture experiments showed that ADSCs survived well in hydrogels. Eight weeks post-operation, the hydrogel/ADSC repair and pure-hydrogel groups showed better defect repair than the blank-control group, and the hydrogel/ADSC repair group displayed articular cartilage surface, as shown by toluidine blue and red “O” bright green staining. The 1.5% GCS/4% DF-PEG hydrogel showed good injectability, self-healing properties, and biocompatibility and provided a good cell proliferation microenvironment. GCS/DF-PEG hydrogels functioned as cell carriers for ADSC transplantation, enabling articular-cartilage defect treatment. The simple operation showed efficacy compared to the blank group. Hydrogel composition and structure should be improved in future for improved cartilage-repair effect.

## Materials and Methods

### Hydrogel Fabrication

#### Preparation of GCS/DF-PEG Hydrogels

GCS powder was dissolved with stirring in deionized water to a concentration of 1.5% (mass/vol) and sterilized using a 0.22 μm filter. Dialdehyde-functionalized PEG powder was dissolved in deionized water, with stirring, and sterilized using a 0.22 μm filter. GCS solution was mixed with an equal volume of sterile dialdehyde-functionalized PEG solution and shaped in a 96-well plate to obtain a GCS/DF-PEG hydrogel, as described (Li et al., 2017). Briefly described as: PEG2000 (3.26 g, 1.63 mmol), 4-formylbenzoic acid (0.98 g, 6.52 mmol), and DMAP (0.050 g) were dissolved in 100 mL of dry THF, followed by the addition of DCC (1.68 g, 8.15 mmol) under a nitrogen atmosphere. The system was stirred at 20°C for 18 h; then, the white solid was filtered. The polymer was obtained as a white solid after repeated dissolution in THF and precipitation in diethyl ether for three times. Upon drying, 3.00 g of dialdehyde-functionalized PEG (DF-PEG) was obtained in 79.8% yield.

#### Electron Microscopy-Based Detection of the Porosity of GCS/DF-PEG Hydrogels

The hydrogel sample prepared above was dehydrated using a vacuum freeze dryer for 24 h, a cross-section was collected, and the sample was cut into 0.5–1.0 mm flakes. The sheet was placed in a vacuum chamber, uniformly coated with a thin layer of gold, and then loaded into a field-emission scanning electron microscope for topographical imaging (Liu et al., 2017).

#### Different Mass Fractions of Aldehyde-Functionalized, GCS/DF-PEG Hydrogel in *in vitro*-Degradation Experiments

Using the methods described above, we prepared a 1.5% GCS (mass/vol) solution and dialdehyde -functionalized PEG solutions with mass fractions of 2, 4, and 8%. Then, 80 μL of GCS solution was mixed with each of the three dialdehyde-functionalized PEG solutions in equal volumes to obtain three different dialdehyde-functionalized PEG-crosslinked GCS/DF-PEG hydrogels. Each group of hydrogels was prepared according to the same specifications, immersed in phosphate-buffered saline (PBS) (pH 7.4, 37°C) and 5% CO_2_, and observed over the course of 4 weeks. For each group, three pieces were collected each week, water droplets were absorbed to the surface of a rubber block with absorbent paper, the mass was measured with a balance, and then used immediately before degradation occurred. After 5, 7, 14, 21, and 28 days, the degradation rate for each group of hydrogels was calculated as follows: degradation rate = (quality before degradation–quality after degradation)/mass before degradation × 100% (Qi et al., 2018).

#### Determination of the Elastic Modulus of Different Dialdehyde-Functionalized PEG-Crosslinked GCS/DF-PEG Hydrogels

The 1.5% GCS solution (mass/vol) was mixed with equal volumes of 2, 4, or 8% dialdehyde-functionalized PEG solution, followed by shaping into cylindrical hydrogel blocks. Subsequently, each hydrogel was cut into three pieces and then each set of hydrogel blocks was placed flat on a measuring table. The position of the measuring head was adjusted, such that it came into close contact with the rubber block, after which the rubber block was slowly pressed (0.01 mm/s). The pressing distance was 20% of the total height. The pressure was measured in real time, and the data were analyzed to draw the force curve and calculate the modulus of elasticity (Sun et al., 2017). The shear-thinning and self-healing properties of the hydrogels are characterized in the supplementary information.

### Isolation and Culture of ADSCs

ADSCs were isolated and cultured as follows. SD rats were sacrificed and placed in 75% ethanol for 10–15 min after cervical dislocation. The subcutaneous adipose tissue in the bilateral inguinal region was excised, visible blood vessels in the tissues were removed, and the tissue was washed two or three times with PBS. The tissue was transferred to a sterile penicillin vial, cut into a paste, 0.1% type-II collagenase and magnetic beads were added, and the tissue was digested by stirring. After 30 min, the digestion was terminated by adding low-sugar Dulbecco’s modified Eagle’s medium (DMEM) containing 10% fetal bovine serum. A filter was used to remove large undigested tissue blocks, the filtrate was centrifuged at 1,700 r/min for 5 min, the supernatant was discarded, and the cells were resuspended in low-glucose DMEM supplemented with 10% fetal bovine serum. The cells were transferred to a culture flask and incubated at 37°C with 5% CO_2_; after 24 h, the culture medium was replaced for the first time, and then the culture medium was changed every other day. The cells were subcultured 1:3 when they reached 80–90% confluency.

### Biosynthesis of Hydrogels on ADSCs

#### Determining the Survival Rate of ADSCs in GCS/DF-PEG Hydrogels

The survival of ADSCs in GCS/DF-PEG hydrogels was evaluated by dead–live staining, using DMEM containing 10% fetal bovine serum. Sterile solutions of 3% GCS (mass/vol) and dialdehyde-functionalized PEG (mass fraction, 4%) were prepared. A suspension of passage-2 ADSCs (10 × 10^9^ cells/L) was mixed with an equal volume of sterile 4% dialdehyde-functionalized PEG solution and transferred into a focusing dish. After allowing the mixture to stand until it completely gelatinized, appropriate medium was added to the confocal dish, which was placed in a 37°C, 5% CO_2_ incubator, and stained with LIVE/DEAD stain on day 1 or 5. The cells were then imaged with a laser confocal microscope.

#### Detecting ADSC Proliferation in GCS/DF-PEG Hydrogels

The CCK-8 Cell Counting Kit was used to evaluate the proliferation of ADSCs in different mass fractions of dialdehyde-functionalized PEG-crosslinked GCS/DF-PEG hydrogels. An aseptic solution of 3% GCS solution (mass/vol) was mixed with suspended ADSCs, and 80 μL of the resulting cell suspension was mixed with an equal volume of 2, 4, or 8% dialdehyde-functionalized PEG. The resulting solution was stirred evenly, and gel-shaped hydrogels were formed in 96-well plates. After fully gelatinizing, 150 μL of DMEM supplemented with 10% fetal bovine serum was added to each well at 37°C, 5% CO_2_ incubator. In the same manner, 80 μL of 1.5% GCS solution (with a cell mass fraction of 1.5%) was mixed with a 2, 4, or 8% dialdehyde-functionalized PEG solution, as done in the aforementioned three experiments. The first, third, fifth, and seventh days were chosen as experimental observation points, using three sets of duplicate wells at each time point in each group. At each time point, the medium in the well was replaced with 150 μL of CCK-8 solution, the plates were incubated for 4 h in a 37°C incubator, and then the CCK-8 solution in each well was transferred to another 96-well plate. The absorbance of each well was quickly measured at 450 nm in the dark using a microplate reader, after which a histogram was obtained and analyzed (Tsukamoto et al., 2017).

### Hydrogel Combined With ADSCs for Repairing Articular-Cartilage Defects in Rats

#### Surgical Operation

Four experimental groups were studied, and five SD rats were randomly assigned to each group (Table 1). Preoperative anesthesia was performed with 10% chloral hydrate. Subsequently, the right knee joint was opened patella valgus by medial patellar approach and thigh pulley, and a full-thickness cartilage defect with a diameter of 2 mm and depth of 2 mm was made with a corneal circumcision at the knee joint. In the experimental group, the ADSCs and hydrogel complex were transplanted into the site by injection; in the conditional-control group, only the hydrogel was transplanted into the defect site by injection; no defect was made in the blank-control group. In the positive-control group, only the skin was cut and no cartilage defect was made. The joint capsule and skin were sutured layer-by-layer using sterile nylon suture.

**TABLE 1:** Experimental group treated by different biomaterials.

**Groups**	**Treatments**
Experimental group	GCS/DF-PEG/ADSCs
Condition control group	GCS/DF-PEG
Blank control group	Defect open-ended
Sham	No cartilage defect

#### CatWalk Joint-Repair Function Evaluation

A catwalk gait analyzer was used to analyze the gait scores of SD rats at 8 weeks following surgery. The average footprint strength and average footprint area were analyzed to evaluate the recovery of limb walking function.

#### Histological Evaluation of Articular Cartilage-Repair Effects

Rats were euthanized 8 weeks after surgery, and knee joint specimens were obtained for gross examination and scored according to the International Rehabilitation Association (ICRS) general observation system (Table 1). The repair area of the truncated defect was intercepted, soaked in 10% paraformaldehyde for 24 h, then decalcified with 4% ethylenediamine tetraacetic acid, decalcified for 45 days, embedded in paraffin, and 4-μm thick sections were prepared. The sections were subjected to staining with hematoxylin and eosin (HE), toluidine blue, and modified red O bright green to observe the regenerative repair of cartilage structures and glycosaminoglycan contents.

### Statistical Analysis

The experimental data were statistically analyzed by SPSS 7.0 software. All the data were recorded in a x ± s format, and the inter-group comparison was based on one-way ANOVA. In the experiment, *P* < 0.05 was in the experiment.

## Results

### General and Electron Microscopic Views of GCS/DF-PEG Hydrogels

**FIGURE 1:**
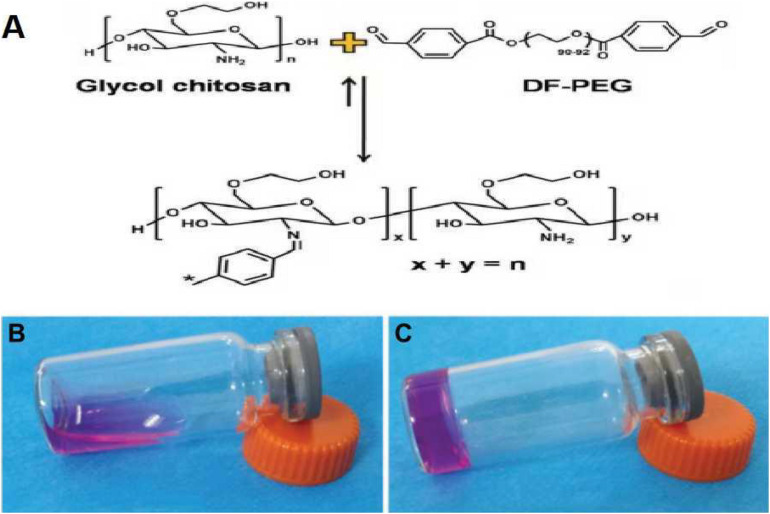
Hydrogel gelation principle and morphology before and after gelation. **(A)** The principle of hydrogel gelation. **(B)** Hydrogel morphology before gelation. **(C)** Hydrogel morphology after gelation.

**FIGURE 2:**
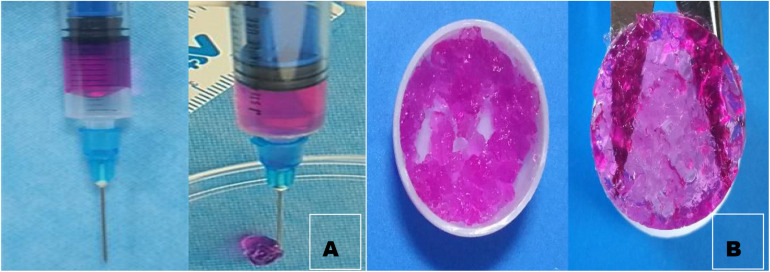
Injectability observation **(A)** and self-healing observation of hydrogel. **(B)** We can see from the graph that the broken hydrogel can self-heal one piece.

As suggested by their molecular and structural formulas, GCS and dialdehyde-functionalized PEG are rapidly gelled by dynamic chemical bonds, which facilitate easy operation. Gel formation was observed 3–5 min after mixing the GCS solution with the dialdehyde-functionalized PEG solution at room temperature (Figure 1). As shown in Figure 2A, the rubber block could be easily passed through a 2 ml syringe, with hydrogels prepared with lower PEG concentrations being easier to push out. As shown in Figure 2B, the broken hydrogel block could be self-healed into a whole piece after being placed in a humid environment for approximately 10 min, with hydrogels prepared with lower PEG concentrations gelling more readily. After the hydrogel was freeze-dried using a vacuum, analysis by scanning electron microscopy showed that it had a porous structure with a pore size of 200–400 μm and good pore connectivity.

### *In vitro* Degradation of GCS/DF-PEG Hydrogels

**FIGURE 3:**
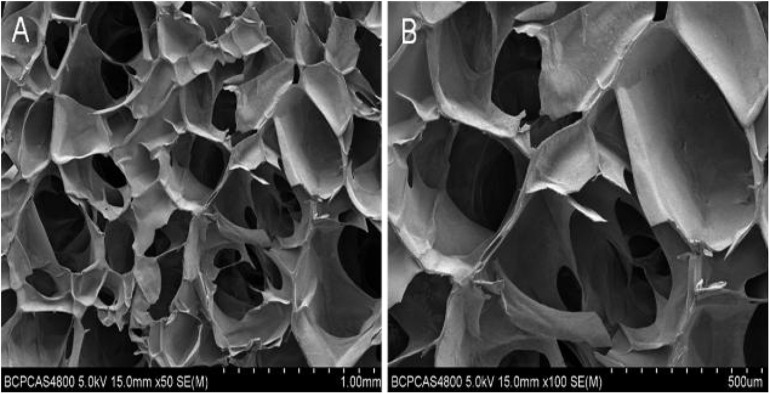
Electron microscopic view of hydrogel. **(A)** Electron microscopic view of hydrogel with low magnification. **(B)** Electron microscopic view of hydrogel with high magnification.

**FIGURE 4:**
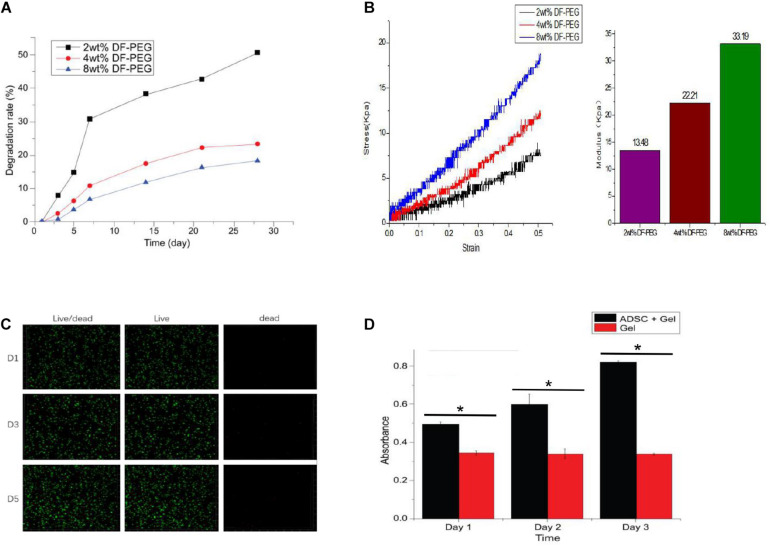
Degradation of hydrogels with different PEG cross-linking concentrations **(A)**, Mechanical testing of different PEG cross-linked hydrogels **(B)**, Inactivated staining of ADSCs in 4% PEG hydrogel **(C)**, and Proliferation assay of ADSCs in 4% PEG hydrogel **(D)**. *P < 0.05.

*In vitro* degradation-rate curves for three groups of GCS/DF-PEG hydrogels with different mass fractions of dialdehyde-functionalized PEG cross-linking are shown in Figure 3. The degradation rate of the gels increased over time, and the degradation rates of the 2, 4, and 8% aldehyde-functionalized PEG hydrogels were 50.67, 23.32, and 18.3% after 4 weeks, respectively. Figure 4A shows that the degradation rate of a hydrogel in the 2%-mass fraction, dialdehyde-functionalized PEG group was significantly faster than those of the other two groups.

### GCS/DF-PEG Hydrogel Elastic Modulus Testing

The mechanical test results were accurately recorded, using the elastic modulus formula with the 2, 4, and 8% dialdehyde-functionalized PEG hydrogel. The elastic moduli were 13.48, 22.21, and 33.19 kPa, respectively. Figure 4B shows that the elastic moduli of the GCS/DF-PEG hydrogels increased with increasing mass fractions of dialdehyde-functionalized PEG.

### Live–Dead Staining of ADSCs in GCS/DF-PEG Hydrogels

The ADSCs in the hydrogels were subjected to live–dead staining and observed by laser confocal microscopy. The living cells showed green fluorescence, and the dead cells showed red fluorescence. Figure 4C shows that the ADSCs in the hydrogel were spherical, and the confocal microscopy results showed that the green fluorescence was stronger than the red fluorescence during the same day, that the number of cells increased on the fifth day compared with the first day, and that the survival rate was above 90%. Thus, the ADSCs survived and proliferated well within the hydrogel.

### Proliferation of ADSCs in GCS/DF-PEG Hydrogels

The CCK-8 method was used to determine the proliferation of ADSCs in GCS/DF-PEG hydrogels with different mass fractions of dialdehyde-functionalized PEG cross-linking. The absorbance values of the control groups did not change significantly (*P* > 0.05) over time. The ADSCs in each experimental group showed significantly increased proliferation (*P* < 0.05). Figure 4D shows ADSC proliferation in the 2%-mass fraction, dialdehyde-functionalized PEG hydrogel. The proliferation rates of ADSCs on the 3rd, 5th, and 7th days were significantly faster in 2%-mass fraction, dialdehyde-functionalized PEG hydrogels than in the 4 and 8% groups (*P* < 0.05). No significant difference was observed in the proliferation rate of ADSCs in the alcohol group (*P* > 0.05).

### Catwalk Gait Analysis

**FIGURE 5:**
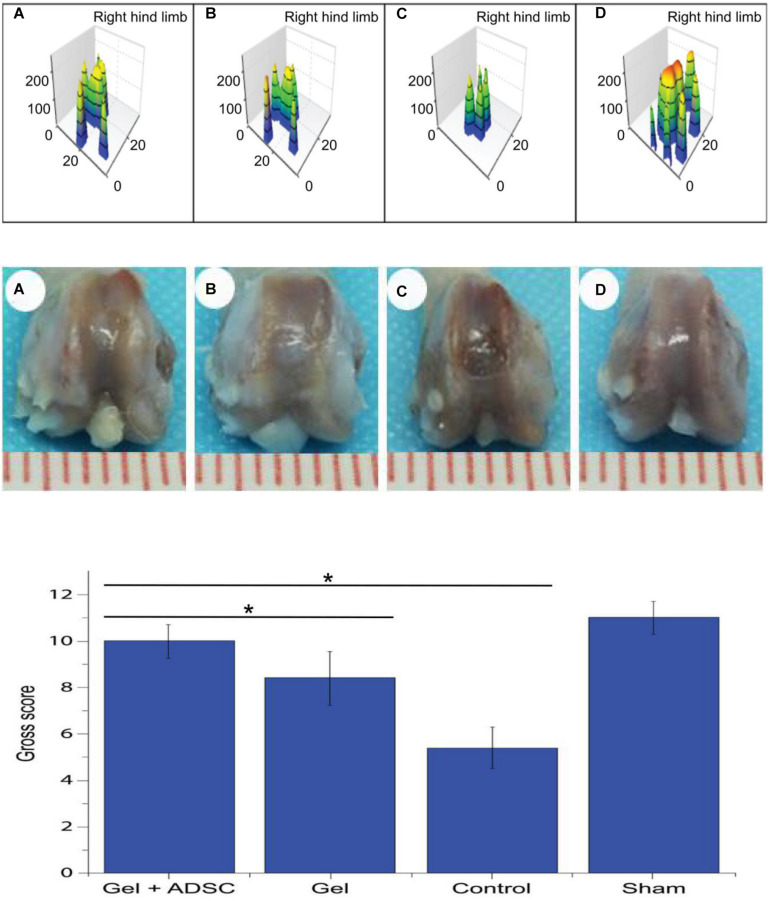
Catwalk gait detection at 8 weeks postoperatively in SD rats. **(A)** ADSCs/hydrogel group; **(B)** pure hydrogel group; **(C)** blank sputum group; **(D)** positive control group (Upper); General view of the 8-week postoperative knee articular cartilage defect repair in SD rats. **(A)** ADSCs/hydrogel group; **(B)** pure hydrogel group; **(C)** blank sputum group; **(D)** positive control group (Middle) and General view score of 8 weeks after repair of knee articular cartilage defects in SD rats (Lower). *P < 0.05.

Catwalk gait analysis (Figure 5A) was performed with the rats at 8 weeks post-operation to evaluate the recovery of post-operative limb functions. The footprint pressure map shows that the postoperative footprint pressure gradually recovered. However, the degree of recovery of the hydrogel-composite ADSC group was significantly better (*P* < 0.05) than that of the simple hydrogel group and blank-control group. This finding indicates that the auto-function recovery of the hydrogel-complexed ADSC group was fastest and that the recovery effect was the best.

### General View

Based on the general appearance (Figure 5A–D), no signs of knee infection, degenerative changes, or inflammation were found. At 8 weeks post-operation, the regenerated and repaired tissue showed a whitish color The repaired tissue in the experimental group was similar to articular cartilage, well fused with adjacent cartilage, and the contour of the femoral block was restored (smooth articular surface without cracks). After repair, the knee joint tissue in the blank-control group was slightly irregular, partially restored to the contour of the femoral block, and the margin of the defect was slightly gapped. In the defect-control group, the contour of the femur block did not recover, the defect area was clearly visible and slightly enlarged, and the knee joint was visible as a whole. The scores of each group were evaluated according to the ICRS general observation-evaluation system (Figure 5C). The following scores were found: ADSCs/hydrogel group (experimental group): 10, hydrogel repair group (conditional-control group): 8.4, blank-control group: 5.4, positive-control group (Figure 5D): 11. The scores in the experimental group were significantly higher than those in the hydrogel-transplantation group and the blank-control group (*P* < 0.05).

### Organizational Evaluation

**FIGURE 6:**
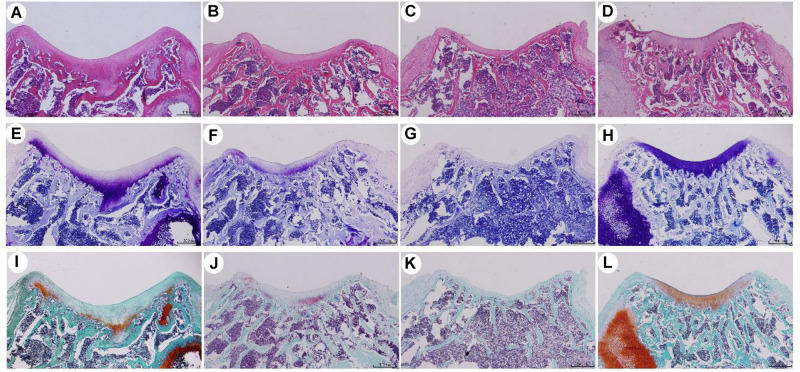
Histological staining of SD rats after knee joint cartilage defect repair for 8 weeks. **(A,E,I)** were ADSCs/hydrogel group; **(B,F,J)** were pure hydrogel groups; **(C,G,K)** were blank group; **(D,H,L)** were positive control groups. **(A–D)** is HE staining; **(E–H)** is toluidine blue staining; **(I–L)** is safranin O staining.

Histological staining of SD rats at 8 weeks after knee joint cartilage-defect repair is shown in Figure 6. In the experimental group, hydrogel-ADSCs were used to repair cartilage defects of the knee joint. After 8 weeks, HE staining showed that the defect area had thicker new tissue regeneration, and the boundary between the regeneration/repair area and the surrounding cartilage was not obvious. Toluidine blue staining showed that the repaired area was filled with a large amount of neonatal cartilage and cartilage-like tissue. Neonatal chondrocytes are similar in structure to normal hyaline chondrocytes, and numerous cartilage lacunae are present in the regenerative area. Improved safranin-O bright-green staining showed a significant amount of repair, based on proteoglycans in the area.

For the conditional-control group, we used hydrogels to repair the cartilage defect of the knee joint. HE staining revealed regeneration with new tissues growing in the defect areas, although the regenerated tissue was thinner than in the experimental group, and the boundary between the regeneration/repair area and the surrounding cartilage was clearly visible. Toluidine blue staining showed that the repaired area was filled with a small amount of new cartilage and cartilage-like tissue, and the cartilage lacuna in the regeneration area was not obvious, when compared with the experimental group. Improved safranin-O bright-green staining showed repair with a low level of proteoglycans in the area. In the defect-setting group, HE staining did not show obvious tissue regeneration in the defect areas, and it was not closely attached to the subchondral bone. Subchondral bone leakage was visible in some areas. Toluidine blue staining showed that the repaired area was filled with fibrous tissue, and the modified safranin was improved. Safranin-O, bright-green staining showed repair and the presence of almost no proteoglycans in the region.

## Discussion

The natural structure of articular cartilage lacks blood vessels, lymphatic vessels, and nerves, resulting in the inability to provide sufficient cells for efficient self-healing of cartilage in the defect area. In this study, from the perspective of tissue engineering, an injectable chitosan hydrogel was used to transplant ADSCs into the articular cartilage-defect area for articular cartilage regeneration and repair. Attempts were made to establish a feasible method for developing osteoarthritis by injection-mode stem cell transplantation to delay articular cartilage injury.

The use of mesenchymal stem cells as cartilage tissue-engineering seed cells for cartilage regeneration/repair *in vitro* and *in vivo* is gradually increasing. Mesenchymal stem cells are widely available and can be extracted from adipose tissue, placenta, bone, deciduous teeth, and synovial tissue, among other tissues, and are therefore referred to as different types of mesenchymal stem cells. Among them, ADSCs have multi-differentiation potential, can be cultured *in vitro*, have low immunogenicity, and can be acquired from various sites in the body (Huang et al., 2013; Chen et al., 2014; de Girolamo et al., 2015; Ansari et al., 2017; Liao et al., 2017; Liu et al., 2017). As well as a high acquisition rate (adipose tissue can provide more ADSCs than bone marrow). The availability of more stem ADSCs (∼5,000 cells per gram of tissue, compared to 100–1,000 bone marrow-derived stem cells) is preferable for many researchers. ADSCs secrete large amounts of cytokines and growth factors, such as hepatocyte growth factor, interleukin-6, macrophage colony-stimulating factor, transforming growth factor beta 1 (TGF-β1), and tumor necrosis factor-alpha (TNF-α), which support tissue remodeling and inhibit apoptosis. Previous reports have shown that ADSCs exhibit multi-line plasticity (multi-directionality) with the ability to differentiate into multiple cell types (derived from three germ layers: mesoderm, endoderm, and ectoderm). Articular cartilage belongs to connective tissue and is evolved in the embryonic mesoderm. *In vitro* studies of ADSCs cultured in the presence of insulin growth factor, bone morphogenetic protein, and TGF-β can express Sox-9, proteoglycan, cartilage oligomeric matrix protein, chondroitin sulfate, and type II collagen. The expression of cartilage matrix-associated protein was increased. A large number of ADSCs was used for cartilage tissue engineering *in vitro* and *in vivo* to obtain satisfactory results (Im, 2016; Tabatabaei Qomi and Sheykhhasan, 2017).

Biomaterials play important roles in cartilage tissue engineering, providing not only a carrier for cells and factors, but also a good microenvironment for cell proliferation, phenotype development, and differentiation, which in turn promotes the regeneration of articular cartilage. In addition, the preservation of biological materials is a focus of tissue engineering researchers. Different forms of biological materials have different properties. At present, the cartilage-repair materials used in tissue engineering include hydrogels, freeze-dried materials, woven materials, electrospun materials, and composite materials. Hydrogel materials have been used in cartilage tissue engineering because of their high water content (similar to articular cartilage) and good biocompatibility. Injectable hydrogels are an important class of hydrogels because they can be easily bolused with syringes. The encapsulated cells can be filled into cartilage defects of any shape; thus, they have large clinical research potential, are widely used in cartilage tissue engineering (Liu et al., 2017), and have supported a major direction for preparing cartilage-repair hydrogel materials in recent years. Injectable hydrogels typically retain large amounts of water, have good permeability to nutrients and metabolites, and exhibit good biocompatibility. They can be administered using minimally invasive procedures and can be used to properly fill irregularly shape defects (Liao et al., 2017). In addition, cells and bioactive molecules can be uniformly incorporated into the hydrogel. Injectable hydrogels, due to their physical properties similar to extracellular matrix, may serve as a suitable platform to support osteoblast survival, proliferation, and differentiation, and promote articular-cartilage tissue regeneration. In addition, self-healing hydrogel has been widely used quite successfully combined with diversified fields, such as magnesium ions mediated fibrocartilaginous interface regeneration (Chen et al., 2020), photothermal therapy of infected full-thickness skin wounds (He et al., 2020), and hepatocellular carcinoma therapy by using pH-responsive drug delivery system (Qu et al., 2017).

The GCS/DF-PEG hydrogels used in this experiment relied on the natural material chitosan (GCS) and the synthetic polymer material PEG (DF-PEG) to form a gel through the dynamic Schiff base chemical bond. It was found experimentally that the injectable hydrogel easily passed through a 26-G syringe and exhibited good injectability. In this study, the injectability and self-healing experiments confirmed that the hydrogel could be easily pushed out of a syringe needle and had strong injectability. After 5–10 min, the scattered hydrogel particles protruding through the syringe aggregate into a whole hydrogel, which is consistent with the strong self-healing properties of hydrogels The presence of chemical bonds, the three-dimensional system of the hydrogel in a state of dynamic equilibrium, and the formation of non-constant chemical bonds between the molecules inside the hydrogel enable a self-healing function. To determine the appropriate method for tissue engineering-hydrogel formulation, based on previous experimental studies, we prepared a 1.5 wt% GCS/2 wt% DF-PEG hydrogel, a 1.5 wt% GCS/4 wt% DF-PEG hydrogel, and a 1.5wt% GCS/8wt% DF-PEG hydrogel. Through degradation experiments, we found that increasing DF-PEG concentrations were associated with a higher modulus of elasticity of the hydrogel. Previous studies have shown that when the hydrogel elastic modulus is 20 KPa, stem cells differentiate into hyaline cartilage and produce extracellular matrix characteristic of cartilage. Based on the experimental results of the comprehensive degradation test and the elastic modulus test of the hydrogel, we used 1.5% GCS/4% DF-PEG hydrogels for the tissue engineering cartilage-repair experiments.

We tested the cytotoxicity and porosity of hydrogels comprised of 1.5 wt% GCS and 4 wt% DF-PEG to further study the feasibility of using it in cartilage-repair applications. The electron microscopy images showed that the GCS/DF-PEG hydrogel had a good pore size of 200–400 um and good void connectivity. Our method of hydrogel production produced ice crystals during lyophilization and, thus, the hydrogel material did not show a true void state. However, it is possible to form numerous microtubule structures inside the hydrogel, which can facilitate the transportation of oxygen and various nutrients in culture medium, and to smoothly discharge harmful substances generated by cell metabolism. The live–dead-staining results showed that the ADSCs grew well in GCS/DF-PEG hydrogel. The spherical three-dimensional growth state in hydrogels is similar to the natural state of ADSCs in the body. This finding also confirmed that the chitosan constituting the hydrogel material has good biocompatibility with the PEG component, and can potentially be used as a cell-transplantation material in tissue engineering.

In this study, GCS/DF-PEG hydrogels containing ADSCs were transplanted into fresh cartilage-defect area by injection. Compared with a traditional stent graft, the operation is simple, and the stent material is not needed in the early stage. Trimming and shaping can adapt to defects of various shapes. Cell transplantation using a hydrogel can facilitate a higher concentration of cells in the cartilage injury area and provide a good microenvironment for cell growth and proliferation. After 8 weeks, Catwalk gait analysis showed that the experimental group had a significant recovery of the limb walking function, compared with the blank-placement group, but it still did not reach the walking function in the normal group. Compared with the simple hydrogel-transplantation group, we found that HE, toluidine blue, and red O staining were darker in the defect area of regenerated tissue, and more cartilage lacuna were seen in the repaired tissue. Soft lower bone leakage was observed in the defect-placement group, and the defect degeneration was characterized by toluidine blue and negative red O. These findings indicated that ADSCs and GCS/DF-PEG hydrogel complexes transplanted into the cartilage defect area showed obvious cartilage regeneration. Comprehensive *in vivo* experiments, gross observations, and measurements of walking function indicated that transplanting a GCS/DF-PEG hydrogel containing ADSCs into a fresh cartilage-defect area can achieve cartilage regeneration and repair, although it was still difficult to generate cartilage (needed for complete regeneration and repair).

## Conclusion

In this study, we explored the use of glycol chitosan/dibenzaldehyde-terminated polyethylene glycol (GCS/DF-PEG) hydrogel to transplant Adipose-derived mesenchymal stem cells (ADSCs) in cartilage regeneration and repair experiments. Although satisfactory results were obtained *in vivo*, there are still some shortcomings. A repair time of 8 weeks in the body is somewhat short, and the long-term effects of the repair method are uncertain. It is hoped that the long-term effect of repair can be detected by prolonging the repair time. Secondly, the mechanism of repairing cartilage damage using this kind of stem cell transplantation method is not clear. It may be that the differentiation of ADSCs into chondrocytes will enable the regeneration of cartilage or promote the cartilage-regeneration ability of host cells. This topic will be the subject of our next experimental study.

## Data Availability Statement

The original contributions presented in the study are included in the article/supplementary material, further inquiries can be directed to the corresponding author/s.

## Ethics Statement

The animal study was reviewed and approved by the treatment of animals conforms to the Guiding Opinions on Treating Animals Promulgated by the Ministry of Science and Technology. Written informed consent was obtained from the owners for the participation of their animals in this study.

## Author Contributions

JY, XJ, QG, SL, and YW carried out the concepts, design, definition of intellectual content, literature search, data acquisition, data analysis, and manuscript preparation. ZW, XL, and XZ provided assistance for data acquisition, data analysis, and statistical analysis. TL, XL, WG, and HR carried out he literature search, data acquisition, and manuscript editing. MC, KL, and XS performed the manuscript review. All authors have read and approved the content of the manuscript.

## Conflict of Interest

The authors declare that the research was conducted in the absence of any commercial or financial relationships that could be construed as a potential conflict of interest.
